# Impact of SARS-CoV-2 vaccination on monoclonal gammopathy of undetermined significance: results from the population-based iStopMM study

**DOI:** 10.1038/s41408-026-01487-x

**Published:** 2026-04-11

**Authors:** Robert Palmason, Elias Eythorsson, Sæmundur Rögnvaldsson, Sigrún Thorsteinsdóttir, Sara Ekberg, Michael Crowther, Elin Ruth Reed, Jon Þórir Oskarsson, Gudrun Asta Sigurdardottir, Thor Aspelund, Brynjar Vidarsson, Pall Torfi Onundarson, Bjarni Agnar Agnarsson, Margret Sigurdardottir, Ingunn Thorsteinsdottir, Signy Vala Sveinsdottir, Isleifur Olafsson, Asdis Rosa Thordardottir, Asbjorn Jonsson, Olafur S. Indridason, Gauti Gislason, Andri Olafsson, Jon Kristinn Sigurdsson, Hlif Steingrimsdottir, Thorir Einarsson Long, Malin Hultcrantz, Brian G. M. Durie, Stephen Harding, Ola Landgren, Runolfur Palsson, Thorvardur Jon Love, Sigurdur Yngvi Kristinsson

**Affiliations:** 1 Landspitali University Hospital, Reykjavik, Iceland; 2 Skane University Hospital, Lund, Sweden; 3 Faculty of Medicine, University of Iceland, Reykjavik, Iceland; 4 Department of Hematology, Rigshospitalet, Copenhagen Denmark; 5 Red Door Analytics AB, Stockholm, Sweden; 6 Akureyri Hospital, Akureyri, Iceland; 7 Myeloma Service, Department of Medicine, Memorial Sloan Kettering Cancer Center, New York, NY USA; 8 Samuel Oschin Comprehensive Cancer Institute, Cedars-Sinai Outpatient Cancer Center, Los Angeles, CA USA; 9 The Binding Site, part of Thermo Fisher Scientific, Birmingham, United Kingdom; 10 Myeloma Program, Department of Medicine, University of Miami, Sylvester Comprehensive Cancer Center, Miami, FL USA

**Keywords:** Epidemiology, Risk factors, Myeloma, Myeloma

Monoclonal gammopathy of undetermined significance (MGUS) is a generally asymptomatic plasma cell disorder and a precursor to multiple myeloma (MM), Waldenström macroglobulinemia, and other lymphoproliferative disorders. The prevalence of MGUS is 3–7% and increases with age, with a progression rate to multiple myeloma and related diseases of about 1–1.5% per year [1, 2].

The factors triggering progression of MGUS remain unclear, though prior infections, such as herpes zoster, hepatitis C, and HIV, have been associated with an increased risk of MGUS [3–5]. These associations may reflect chronic immune stimulation that persists in some infections and could influence MGUS development or progression.

Concerns have been raised about whether vaccinations could potentially trigger malignant growth, particularly the progression of plasma cell disorders. To our knowledge, there is no evidence from studies evaluating the possible effects of vaccination on MGUS or other malignant precursors. It is therefore of public health importance to critically address these concerns in a systematic study.

This study aimed to assess whether vaccination of individuals with MGUS or smoldering multiple myeloma (SMM) is associated with changes in M protein levels over time compared with the pre-vaccination period. The massive COVID-19 immunization campaign provided a unique opportunity to evaluate this in a longitudinal setting in individuals vaccinated within the same period and with little or no prior immunity. We utilized data from the ongoing Iceland Screens Treats or Prevents Multiple Myeloma (iStopMM) [6] study, a prospective nationwide study in which more than 75,000 individuals were screened for MGUS, using capillary zone electrophoresis (CZA) and serum free light chain (FLC) assays (ClinicalTrials.gov identifier: NCT03327597). Immunofixation electrophoresis was performed on samples with clear or suspected M protein bands on CZE, and/or abnormal FLC results. Individuals with suspected MGUS entered a randomized trial: Arm 1 received no study follow-up, while Arms 2 and 3 underwent routine or intensified monitoring. Annual serum protein electrophoresis (SPEP) was performed except in low-risk MGUS in arm 2. Participants with M protein >30 g/L, FLC ratio >100, or light-chain MGUS were excluded.

Iceland’s SARS-CoV-2 vaccination program began in December 2020 and achieved >90% uptake among individuals ≥40 years by February 2022. Vaccine type and number of doses were obtained from the Icelandic Directorate of Health [7].

M protein concentrations were measured longitudinally according to trial arm, typically annually. Vaccination status was linked to each measurement (before/after the first dose, dose number, and vaccine type). Linear mixed-effects models with patient-specific intercepts and slopes were used to assess the association between vaccination and M protein concentration, adjusting for sex and calendar year, with attained age as the underlying time scale. For graphical display, attained age was modeled using restricted cubic splines. M protein values were log-transformed for modeling and back-transformed for reporting. If M protein was identified on IFE but not CZE, its value was set at 0.1 g/L.

A total of 75,422 individuals were screened, identifying 3059 with MGUS. After excluding participants in Arm 1, 2038 individuals had measurable M protein, of whom 1814 (89%) received at least one SARS-CoV-2 vaccination and formed the study cohort. IgG/IgA MGUS was most common (*n* = 1231, 67.9%), followed by IgM (*n* = 360, 19.9%) and biclonal MGUS (*n* = 156, 8.6%). Additionally, 67 individuals (3.7%) were unclassifiable. Median baseline M protein concentration was 0.55 g/L (interquartile range: 0.1–2.1), with 6094 M protein measurements recorded over a median follow-up of 2.3 years (range 0.5–5.5 years).

Among vaccinated individuals, 98.9% received ≥2 doses and 26.2% received 3 doses of vaccine. Median age at first vaccination was 71 years (range 42–98), and 53.9% were male. Pre-vaccination M protein measurements were available for 1627 participants (4520 samples) and post-vaccination data for 1143 individuals (1574 samples), with a median of 10.9 weeks (range 0.1–51) between vaccination and sampling.

Assuming a linear trend over time, the annual increase in M protein concentration before and after vaccination was comparable. The increase was 1.0% (95% CI: 0.2–1.9) before vaccination compared to 1.2% (95% CI: 0.3–2.1) after vaccination (Fig. 1A). When stratified by sex, the annual increase was 1.9% in males (95% CI: 0.8–3.1%) and 0.2% in females (95% CI: −1.0 to 1.4%), with no significant difference between sexes (*p* = 0.16). We also found the results to be consistent across immunoglobulin isotypes. Annual M protein changes before and after vaccination were similar for IgG/IgA (1.2% vs. 1.3%, *p* = 0.585), IgM (1.7% vs. 1.6%, *p* = 0.825), and biclonal MGUS (2.5% vs. 2.2%, *p* = 0.657), with no significant difference between the groups (*p* = 0.514; Fig. 1B). There was no effect of the number of vaccine doses on M protein levels, with similar findings after one, two, and three doses. Results were likewise consistent across vaccine types, with no meaningful differences observed between the vaccines used (Table 1 and Fig. 1C). When analyzing the possible effect of time between vaccination and M protein measurement, no significant difference in M protein concentration was observed immediately after vaccination compared to up to one-year post-vaccination (*p* = 0.850; Fig. 1D). Among the 121 individuals who remained unvaccinated and had follow-up testing during the study period, the estimated annual increase in M protein concentration was 1.1% (95% CI: −1.9 to 4.1%), similar to the overall trend observed in the whole study population.

**Fig. 1:**
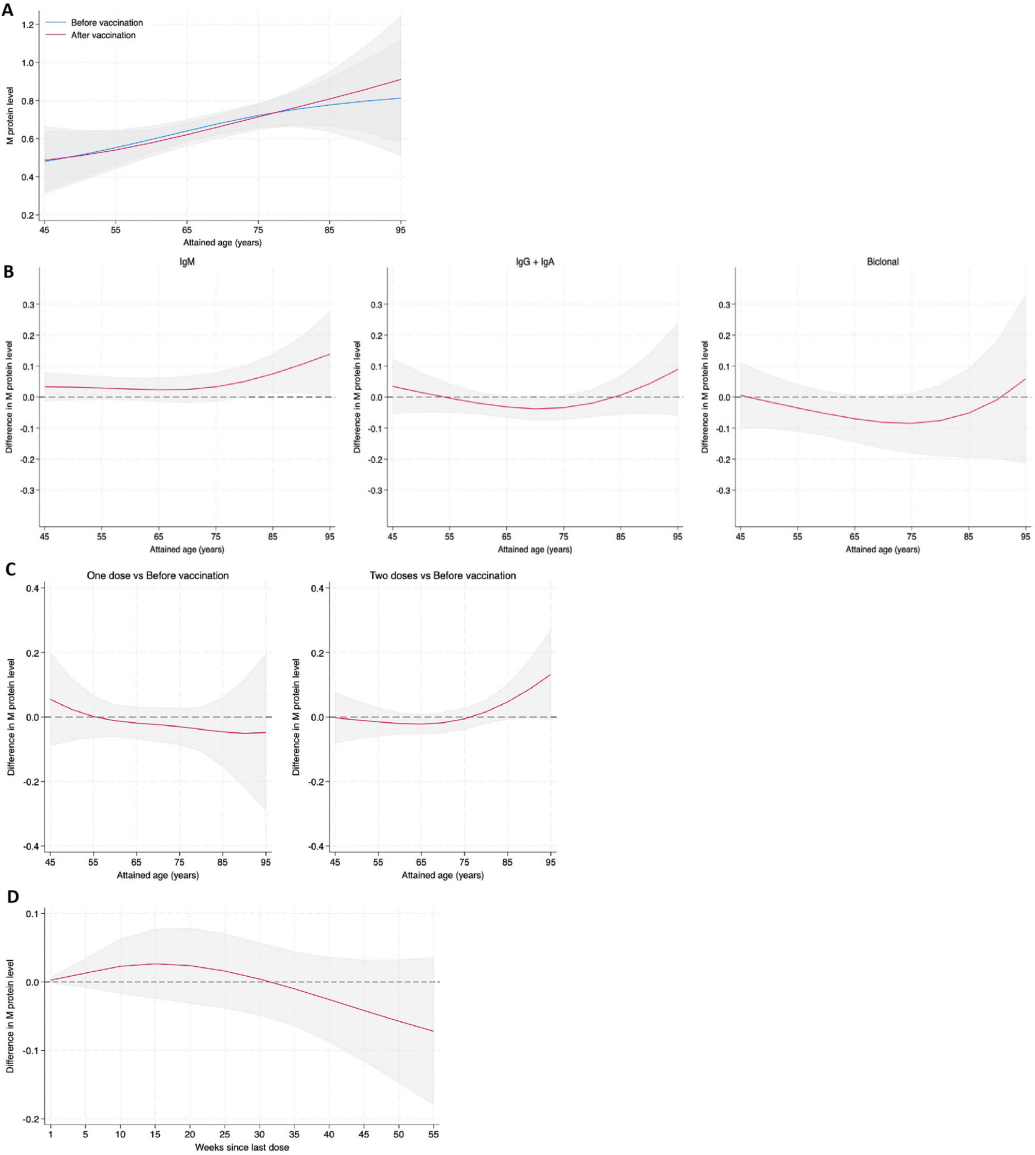
Changes in M protein concentration before and after SARS-CoV-2 vaccination. **A** Predicted M protein levels (g/L), with 95% confidence intervals, before and after SARS-CoV-2 vaccination, shown at different ages. Predictions are based on a model adjusted for sex and calendar year and averaged over the observed distributions of these covariates. **B** Marginal differences in M protein levels (g/L) with 95% confidence intervals at different ages, before and after SARS-CoV-2 vaccination, divided by isotype (IgM, IgG + IgA, and biclonal). Predictions are based on a model adjusted for sex and calendar year and averaged over the observed distributions of these covariates. **C** Marginal differences in M protein levels (g/L) with 95% confidence intervals shown at different ages, before and after one (left panel) and two (right panel) doses of SARS-CoV-2 vaccines. Predictions are based on a model adjusted for sex and calendar year and averaged over the observed distributions of these covariates. **D** Difference in M protein levels (g/L) with 95% confidence intervals before and after SARS-CoV-2 vaccination, by time since last dose of vaccine was administered. Predictions are based on a model adjusting for age, sex, and calendar year, with marginal estimates averaged over the observed distributions of these covariates.

**Table 1:** Relative change per year of M protein concentration in individuals with MGUS, before and after SARS-CoV-2 vaccination.

	All		Males		Females	
	*N* obs / *N* ind.	Exp (*β*) (95% CI)	*N* obs / *N* ind.	Exp (*β*) (95% CI)	*N* obs / *N* ind.	Exp (*β*) (95% CI)
Vaccinated						
Before vaccination	4520/1627	1.00	2366/867	1.00	2154/760	1.00
After vaccination	1574/1143	0.99 (0.96, 1.03)	866/521	1.00 (0.96, 1.04)	708/622	0.98 (0.93, 1.04)
Number of doses						
Before vaccination	4520/1627	1.00	2366 / 867	1.00		1.00
1	279/261	0.96 (0.90, 1.03)	141/134	1.00 (0.92, 1.08)	138/127	0.93 (0.84, 1.03)
2	1128/886	1.00 (0.96, 1.04)	620/483	1.01 (0.96, 1.06)	508/403	0.98 (0.92, 1.05)
3	167/138	1.03 (0.94, 1.12)	105/90	0.97 (0.88, 1.07)	62/48	1.14 (0.98, 1.32)
Type of vaccine						
Before vaccination	4520/1627	1.00	2366/867	1.00	2154/760	1.00
Tozinameran	927/672	0.97 (0.93, 1.02)	510/359	1.01 (0.96, 1.07)	417/313	0.93 (0.87, 1.00)
Vaxzevria	510/387	1.00 (0.95, 1.07)	293/226	1.00 (0.94, 1.07)	218/161	1.02 (0.93, 1.11)
Elasomeran	116/83	1.05 (0.95, 1.17)	53/38	0.93 (0.81, 1.06)	63/45	1.18 (1.01, 1.38)
Jcovden	21/19	1.11 (0.84, 1.48)	11/9	1.00 (0.70, 1.42)	10/10	1.26 (0.80, 1.98)
Combination of vaccines						
Before vaccination	4520/1627	1.00	2366/867	1.00	2154/760	1.00
First dose						
Tozinameran	78 / 74	0.92 (0.82, 1.03)	32/30	0.94 (0.80, 1.10)	46/44	0.91 (0.77, 1.07)
Vaxzevria	170 / 158	0.96 (0.89, 1.04)	95/92	1.00 (0.91, 1.11)	75/66	0.91 (0.79, 1.04)
Elasomeran	10 / 10	1.17 (0.88, 1.58)	3/3	1.37 (0.82, 2.28)	7/7	1.14 (0.78, 1.68)
Jcovden	21 / 19	1.11 (0.84, 1.48)	11/9	1.00 (0.70, 1.42)	10/10	1.26 (0.80, 1.97)
Second dose						
Tozinameran ×2	686/535	0.99 (0.95, 1.04)	375/284	1.03 (0.97, 1.10)	311/251	0.94 (0.87, 1.02)
Vaxzevria ×2	340/273	1.03 (0.97, 1.10)	197/162	1.00 (0.92, 1.08)	143/111	1.07 (0.97, 1.19)
Elasomeran ×2	82/62	1.00 (0.88, 1.13)	36/27	0.82 (0.70, 0.97)	46/35	1.18 (0.98, 1.41)
Other^1^	20/16	0.65 (0.50, 0.84)	12/10	1.08 (0.78, 1.48)	8/6	0.32 (0.21, 0.49)

Exponentiated beta coefficients (Exp(β)) with 95% confidence level (CI) from mixed linear models of log M protein (g/L) with random intercepts and slopes. All estimates were adjusted for attained age, sex, and calendar year. The estimates are also presented separately for males and females.

*N* obs/*N* ind. = number of M protein observations (obs.) and contributing individuals (ind.). Individuals may contribute multiple observations and appear in more than one group.

^1^Vaxzevria dose 1 and Tozinameran as dose 2, or Jcovden as dose 1 and Tozinameran or Elasomeran as dose.

In the study cohort, 134 individuals (7.4%) met criteria for SMM [8] during follow-up, contributing 810 M protein measurements. In this subgroup, the estimated annual change in M protein concentration was 2.1% before vaccination (95% CI: −3% to 7%) and 1.9% after vaccination (95% CI: −3% to 7%), with no significant difference between the slopes (*p* = 0.559).

Our findings provide evidence that SARS-CoV-2 vaccination is not associated with an accelerated increase in M protein levels in individuals with MGUS and SMM. The annual increase in M protein concentration was essentially unchanged following vaccination, irrespective of the number of doses administered. The results were also stable across vaccine platforms, including both mRNA and adenoviral vector vaccines. Importantly, the small group of unvaccinated individuals showed the same M protein trajectory as vaccinated participants. The absence of any signal was consistent across MGUS subtypes and in SMM. Also, no change in M protein levels was observed either immediately after vaccination or during the subsequent year.

Our finding of no change in M protein levels following vaccination suggests that transient immune activation after SARS-CoV-2 vaccination does not alter the behavior of the premalignant plasma cell clone. To our knowledge, this is the first study to evaluate whether vaccination against SARS-COV-2, or any other pathogen, affects the development of MGUS or SMM, and these results may be valuable in addressing vaccine hesitancy.

Long-term follow-up studies of individuals with MGUS [1, 8] have shown that M protein levels tend to increase over time, making it essential to compare the post-vaccination slope with each individual’s pre-vaccination trajectory rather than rely solely on absolute changes in M protein concentration. The biological basis for the gradual rise in M protein concentration and its link to progression remains incompletely understood. MGUS is associated with persistent antigenic stimulation, a disrupted marrow microenvironment, and immune dysregulation [9, 10]. Although some mature B-cell neoplasms are linked to infections such as Epstein–Barr virus, hepatitis C, and Helicobacter pylori [11–13], evidence for a similar association with MGUS or MM is limited. While most M proteins are non-functional, some target microbial antigens [14], and chronic viral infections, such as hepatitis B, hepatitis C, and HIV, have been linked to MGUS and MM, likely through prolonged immune stimulation [4, 5]. However, vaccines activate the immune system only transiently [15], and we did not observe any increase in M protein levels beyond their expected background trend, irrespective of vaccine type.

The primary strength of this study is its large, prospective, and nationwide design, uniform laboratory testing, complete vaccination registry data, and large number of longitudinal M protein measurements. This study experienced low dropout rates, and most individuals with a confirmed diagnosis of MGUS had M protein measurements performed after SARS-CoV-2 vaccination.

Our study has some limitations, the principal one being that only vaccinations against SARS-CoV-2 were included. Because the study period covered only the first three vaccine doses, later booster doses could not be assessed. However, the consistency across doses 1–3 makes it unlikely that later boosters would have had a different effect. Furthermore, the study relied on increasing M protein concentration as a surrogate marker for progression, rather than clinical progression, which is relevant because clinical progression to MM can occur even with modest M protein changes. Finally, the study period was relatively short, with the cut-off date coinciding with the cessation of extensive PCR testing for SARS-CoV-2 in Iceland.

In conclusion, this large, prospective population-based screening study, including more than 75,000 screened individuals and 1814 vaccinated persons with MGUS and 134 with SMM, showed no evidence that SARS-CoV-2 vaccination affects M protein dynamics. Annual increases in M protein levels were small and similar before and after vaccination, across sexes, isotypes, vaccine types, and dose numbers. These findings provide reassurance that SARS-CoV-2 vaccines do not adversely affect M protein dynamics in individuals with MGUS and SMM.

## Data Availability

Current approvals do not allow for the sharing of the underlying study data. However, data may be shared with other investigators pending the review of the iStopMM investigators and the Icelandic Bioethics Committee.
